# Public consultation changes guidance on the use of health‐care interventions. An observational study

**DOI:** 10.1111/hex.12476

**Published:** 2016-06-17

**Authors:** Bruce Campbell, Jeffrey Tabiri‐Essuman, Helen Gallo, Vassilia Verdiel, Lakshmi Mandava, Mohamed Ansaf Azhar, John Powell

**Affiliations:** ^1^ Interventional Procedures Advisory CommitteeNational Institute for Health and Care ExcellenceLondonUK; ^2^ Royal Devon and Exeter HospitalExeterUK; ^3^ Centre for Health Technology EvaluationNational Institute for Health and Care ExcellenceLondonUK; ^4^ Sandwell Metropolitan Borough CouncilOldburyUK; ^5^ Health Experiences Research GroupNuffield Department of Primary Care Health SciencesUniversity of OxfordOxfordUK

**Keywords:** guidance, health policy, public consultation

## Abstract

**Objectives:**

To investigate the responses to public consultation on draft guidance on interventional procedures (IP) for the UK National Health Services, and the changes made as a result of consultation.

**Design:**

Retrospective review of responses received during public consultation for 183 pieces of draft guidance, and subsequent changes made.

**Setting:**

The National Institute for Health and Care Excellence in the UK. Guidance produced December 2009–December 2014.

**Main outcome measures:**

Numbers (%) of public consultations receiving responses, and resulting changes made to draft guidance.

**Results:**

Responses were received during 159 (86.9%) periods of public consultation, from a total of 853 people or organizations (median number per consultation 3; range 0–82; interquartile range 1–5). Changes were made to draft guidance following 136 (74.3%) consultations. These changes were to the category (2.7%) or wording (8.7%) of the main recommendation; to other recommendations (about consent, patient selection, training and future research) (31.1%); and to other sections of guidance (description of the procedure and of the evidence on its efficacy and safety) (70.5%). Additional published evidence was proffered for 22.4%. Health‐care professionals or their specialist societies were the most frequent responders to consultation (68.8%), patients or patient organizations accounted for 22.4% and medical device companies accounted for 8.8%.

**Conclusions:**

This study shows substantial engagement with public consultation and frequent changes made to draft guidance as a result. These findings are likely to be relevant to other areas of health‐care and national policymaking that seek to be responsive to their stakeholders.

## Introduction

Public consultation has become common practice across a wide range of policymaking.^1^ Consultation allows engagement with stakeholders and other interested parties and increases the level of transparency of decision making about matters which may have significant impact on large numbers of people, but the value of participatory activities may depend on how they are conducted and how the responses are used.^2^ There is a common perception that consultation is often neither genuine nor influential, being used to support decisions which have already been made and having little or no impact on them.^3^ The UK Government stipulates that its departments should ‘explain what responses have been received and how these have been used in formulating policy’ and there are clear guidelines for the conduct of public consultations in other countries, but nevertheless scepticism seems to be widespread.^4^, ^5^ Public consultation can have a variety of functions, including gathering opinions, seeking information, identifying unintended consequences or practical problems, checking the relevance and accuracy of draft documents, enhancing the accountability and transparency of policies, and potentially increasing professional and public buy‐in to the final recommendations.^6^

In making policy decisions about health‐care, public consultation is not in common use worldwide and there is a dearth of empirical literature about its influence in shaping decisions and recommendations about health care.^7^ A recent review concluded that there is a lack of robust evidence on the impact of public involvement in health policy.^7^ There have been reports on ways of involving patients and the public in discussions and decision making about health‐care policy and interventions, but not about open public consultation on draft guidance or recommendations for health services.^8^, ^9^, ^10^, ^11^, ^12^, ^13^, ^14^, ^15^, ^16^

The Interventional Procedures (IP) programme at the National Institute for Health and Care Excellence (NICE) produces guidance on new procedures which are entering use in the UK health services, based on evidence about their efficacy and safety.^17^ It also considers procedures which are not new but for which uncertainties have arisen about their safety or efficacy. There is no consideration of cost. Procedures are notified through an unlimited variety of sources.^18^ Consultation takes place at various stages of the development of guidance. Importantly, draft guidance is available on the World Wide Web for a 4‐week period of public consultation. The objective of this study was to determine the frequency and nature of changes made to guidance as a result of responses to public consultation, so illuminating an aspect of health technology assessment and policymaking which has not been well described before.

## Methods

In this study, we wanted to examine the frequency and nature of changes resulting from having a period of public consultation on draft NICE IP guidance. Our index event in this study was therefore ‘a period of public consultation’. This study was conducted in June–September 2015. We identified 200 consecutive pieces of draft NICE IP guidance which had been consulted upon between December 2009 and December 2014. From this 200, we selected all draft guidance documents which were having their first period of public consultation. Documents which were being consulted on for a second or subsequent time were excluded (17 were subject to more than one public consultation – 13 had two consultations, 1 had 3 and 1 had 4). The reason for excluding them was that further consultations are usually only carried out when a significant change has been made to the guidance as a consequence of an initial consultation, and therefore, a second or subsequent consultation period is qualitatively different because public responses have already been taken into account and the guidance amended. This left a study sample of 183 draft guidance documents with a first period of public consultation, covering a diverse range of IP from across the clinical spectrum.

Data were extracted from the following sources:

1. The IP Programme's planning database, which stores procedure titles, consultation dates, publication dates, draft main recommendations and final main recommendations.
2. The tables of consultation comments used by the NICE IP Advisory Committee, which show the numbers of comments and the number and types of consultees as well as the changes made by the committee to the various sections of the guidance.
3. The text of the draft guidance and the final guidance, to check and characterize the precise nature of the changes which were made.

All data were extracted and entered into a Microsoft Access database, which was subsequently exported into SPSS 15 for descriptive statistical analysis, mainly calculating proportions and percentages to describe consultees and the changes made for each part of the guidance.

The methods NICE uses for producing draft guidance for public consultation are as follows. For each procedure notified, an eligibility assessment is carried out and a scoping document is then prepared and reviewed by the IP Advisory Committee. Subsequently, an overview is prepared which includes summaries of the published evidence on the procedure, and written comments from medical specialists and from patients, in response to structured questions. Based on this information and advice, the committee drafts guidance on the procedure which includes recommendations (Box 1) and a series of other structured sections (Box 2).

Box 1 **Recommendations used in NICE Interventional Procedures guidance**

**Section 1: Main recommendation – four possible categories**

1. *Use with normal (standard) arrangements for clinical governance, consent and audit*. The evidence shows that the procedure works well enough and is safe enough for clinicians to use as part of their normal practice, with the usual local policies for clinical governance, patient consent and audit.
2. *Use with special arrangements for clinical governance, consent and audit*. The evidence on safety and/or efficacy leaves significant uncertainties. Hospitals need to ensure that their facilities and risk management arrangements are adequate. There is a greater need for explicit information for patients as part of obtaining their consent. Follow‐up and critical review of outcomes are especially important.
3. *Use only in research*. The procedure should only be performed in the context of formal research studies. Guidance specifies the most important outcomes which need to be elucidated.
4. *Do not use*. The evidence suggests the procedure is not effective, and/or it has unacceptable safety risks.

**Other recommendations**

These may include recommendations about the following:

1. consent – specific matters of special importance which patients should be told
2. patient selection – usually specifying the types of specialists who should be involved
3. training and/or experience of clinicians doing the procedure
4. submission of data on all patients to a specified register
5. further research – types of studies and outcomes needed to resolve uncertainties about the safety and/or efficacy of the procedure.

Box 2 **Structured sections of NICE Interventional Procedures guidance (which follow recommendations in Section 1)**

*Section 2: Indications and current treatments*. A brief description of the conditions the procedure is intended to treat and the current treatment options.

*Section 3: The procedure*. This is brief and intended only to describe to non‐specialists what happens during the procedure: it is not a detailed description for specialists.

*Section 4: Efficacy*. A summary of the most relevant efficacy data in the peer‐reviewed studies considered by the committee.

*Section 5: Safety*. A summary of the most relevant safety data considered by the committee (including non‐peer‐reviewed data, for important additional safety outcomes).

*Section 6: Committee comments*. Any particularly important comments which the committee wishes to highlight, such as the potential of a procedure to benefit a needy group of patients, special difficulties in interpreting the evidence, or uncertainties posed by the use of different or evolving devices for performing the procedure.

The draft guidance document is placed on the NICE website for a period of open public consultation for 4 weeks. The following organizations are forewarned of the public consultation period and are contacted (and sent a link to the document) when consultation begins. Although these organizations are encouraged to take part, there is no compulsion on them to do so.

1. Professional medical organizations involved in the procedure
2. Clinicians nominated by their specialist organizations, who have provided advice during the assessment process
3. National patient organizations identified as representing patients who might receive the procedure
4. Medical device manufacturers whose devices are intrinsic to the procedure
5. The person who notified the procedure to NICE
6. Any person known to have been closely involved in the development of the procedure
7. Any clinicians, patients and any other persons or groups who have registered an interest in the procedure.

Any of these people or organizations may submit responses to the public consultation. In addition, anyone else who wishes to do so may respond, from within or outside the United Kingdom.

Responses to this period of public consultation may be submitted via the NICE website, by email, fax or post. The maximum length of response is 20 pages. Consultees are asked to make responses against the six numbered sections of the guidance. If their responses fail to do this, then the NICE team identifies the relevant sections. The response of each consultee to each section of the guidance is allocated a comment number. The comments are formatted anonymously, with a number, the type of consultee (e.g. health‐care professional, patient, manufacturer), the section of the guidance to which they refer, the full text of the response to that section of the guidance, and commentary from the NICE analyst. This is presented to the committee, which considers each numbered comment and decides whether or not to make changes to the guidance. The committee has 25 members (see Box 3). A selected committee member, who is not a specialist in the relevant field, leads the committee through consideration of each numbered comment, assisted by the committee chair. Each comment is discussed and a decision is made about whether to make any changes. Several comments about the same point may be discussed together.

Box 3 **Membership of the Interventional Procedures Advisory Committee**

The committee is made up of 25 members who are independent of NICE. All members are appointed following public advertisement apart from the Medical Director (Devices) of the Medicines and Healthcare products Regulatory Authority (MHRA).

The membership includes the following:

1. clinicians who carry out interventional procedures (appointed to represent the range of expertise required for the procedures, and regularly reviewed)
2. two lay members who are familiar with the issues affecting patients and carers
3. experts in the evaluation of health care
4. a Chief Executive of an NHS trust
5. a Medical Director of an NHS trust
6. a General Practitioner
7. a nurse
8. a representative from the medical device industry
9. a member with special knowledge of patient safety issues.

After the committee meeting at which public consultation comments are considered, the resulting guidance is considered by NICE's Guidance Executive group, made up of NICE executive directors, guidance centre directors and the communications director. It may also be sent, on request, for a pre‐publication check (known as resolution) to all the people listed above, who have the opportunity to challenge any aspect of the final guidance on the basis of factual inaccuracy or a failure of NICE to follow its published processes.

## Results

There were no missing data. Consultation responses were received for 86.9% (159/183) of the included consultations, from a total of 853 people or organizations. On 24 occasions, no responses were received during the period of public consultation. The categories of the 853 people or organizations who responded were as follows: NHS professionals 40.6% (346/853), individual patients 19.7% (168/853), clinical specialists who had advised NICE about the procedure 11.3% (96/853), medical device companies 8.8% (75/853), specialist medical/surgical societies 7.9% (67/853), private health sector professionals 5.2% (44/853), overseas health‐care professionals 4.0% (34/853) and patient organizations 2.7% (23/853). The median number of consultees who sent responses was 3 per consultation (range 0–82; IQR 1–5) and the median number of comments per consultation was 11 (range 0–457; IQR 4–24). The three pieces of guidance that received the most responses also illustrate the diverse topics covered by the programme: percutaneous venoplasty for chronic cerebrospinal venous insufficiency for multiple sclerosis (82 consultees and 457 comments); transcutaneous neuromuscular electrical stimulation for oropharyngeal dysphagia (62 and 288); and mechanical clot retrieval for treating acute ischaemic stroke (33 and 219).

Overall, the number of consultations from which the responses resulted in a change to the draft guidance was 74.3% (136/183). With regard to the nature of these changes, consultation responses led to changes in the provisional recommendations section for 38.3% (70/183) of the draft guidance documents. A change was made to the category of the main provisional recommendation in 2.7% (5/183). These changes were made to ‘research only’ recommendations on two occasions (one changed to ‘normal arrangements’ and one changed to ‘special arrangements’ for some indications); to ‘special arrangements’ recommendations on two occasions (both changed to ‘normal arrangements’); and to one guidance with a recommendation of ‘normal arrangements’ and ‘special arrangements’ for different indications to ‘special arrangements’ only. Changes were made to the wording (but not the category) of the main recommendation in 8.7% (16/183). Other parts of the recommendations section were changed in response to 31.1% (57/183) consultations: Table 1 shows which recommendations were changed and the types of changes which were made.

**Table 1:** The percentage (number) of occasions on which changes were made to provisional recommendations in draft NICE Interventional Procedures guidance

Type of recommendation	Type of change	%	Number
Main recommendation	Major change – to category	2.7	5/183
	Wording amended	8.7	16/183
	Total	11.4	21/183
Consent	Wording amended	4.9	9/183
	Section added	0	0
	Section removed	1.1	2/183
	Total	6.0	11/183
Patient selection	Wording amended	13.7	25/183
	Section added	2.7	5/183
	Section removed	0.5	1/183
	Total	16.9	31/183
Training and experience	Wording amended	4.4	8/183
	Section added	1.1	2/183
	Section removed	0	0
	Total	5.5	10/183
Data collection/registers	Wording amended	1.6	3/183
	Section added	4.4	8/183
	Section removed	0	0
	Total	6.0	11/183
Further research	Wording amended	10.4	19/183
	Section added	2.2	4/183
	Section removed	1.1	2/183
	Total	13.7	25/183
Total: change to any of the recommendations above (Note that changes were made to more than one section of the recommendations on a number of occasions)		38.3	70/183

The consultation responses resulted in changes to other sections of the guidance (apart from the recommendations) following 70.5% (129/183) consultations. These are shown in Table 2.

**Table 2:** Percentage and number of occasions on which changes were made to sections of the guidance, apart from the recommendations

Section of guidance	%	Number
Section 2. Indications and current treatments	36.7	67/183
Section 3. The procedure	44.8	82/183
Section 4. Efficacy	18.0	33/183
Section 5. Safety	14.2	26/183
Section 6. Committee comments		
Wording amended	6.0	11/183
Section added	19.1	35/183
Section removed	0.5	1/183
Total	25.7	47/183
Total: change to any of the sections listed above (Note that changes were made to more than one section of the guidance on a number of occasions)	70.5	129/183

In 22.4% (41/183) of public consultations, responses were received which proffered additional empirical studies that were subsequently added to the procedure overview document, which is used by the committee as a basis for its deliberations. These changes were in addition to those made to the draft guidance, listed above. Typically, the additional studies were ones published after NICE's original literature review, which had also been retrieved by a routine updated search, but occasionally undiscovered studies were identified. The additional studies were considered by the committee, alongside the evidence already reviewed, to decide whether their findings should change any aspect of the draft guidance.

## Discussion

The finding that responses were received on 86.9% of occasions provides evidence of substantial engagement with the process of public consultation. Subsequent changes to guidance in 74.3% of occasions show that public consultation can lead to tangible changes in evidence‐based public policy, in this case national guidance on IP. The category of main recommendation was changed in just 2.7%, but its wording was altered in 8.7%: this is in tune with the relatively small proportion of responses which challenged the main recommendation, compared with those about other sections of the draft guidance. By contrast, many responses were about minor changes to the wording of other parts of the guidance. Getting the descriptive parts of guidance into a form which addresses even minor concerns of stakeholders is arguably influential in maximizing its credibility and therefore its impact.

A limitation of this study was the lack of detail about the precise nature of the responses to public consultation, the specific changes which were made, and the reasons why changes were made (or not made). We plan further qualitative work to examine the precise nature of responses to consultation, in particular which types are most useful and influential. Discovering the committee's reasoning for making, or not making, changes is not possible in retrospect because that is done by discussion, which is often complex. This study addresses the impact of public consultation in relation to only one of NICE's decision‐making committees. However, committee chairs and members are briefed in detail on how they should consider responses to public consultation, and senior members of NICE attend the various different committees to observe their work and to monitor adherence to proper process. The findings of this study are therefore likely to be broadly representative of the way that NICE handles consultation comments across its many areas of work, and we believe it has relevance for organizations making public policy more broadly.

The use of open public consultation in producing guidance on health‐care interventions in the way NICE does is unusual, and this report on its influence is unique. Other publications have described various ways of involving patients and the public in making decisions about specific health‐care issues, but they have not addressed open public consultation as a regular feature of producing guidance for health services.^8^, ^9^, ^10^, ^11^, ^12^, ^13^ There have been some wide ranging publications about the principles and possibilities for involving the public in decisions about health‐care policy, but we have identified no reports on the use of open public consultation and the influence which it has on the production of guidance about health‐care interventions.^8^, ^9^, ^10^, ^11^, ^12^, ^13^, ^14^, ^15^, ^16^ There has been some evidence of public consultation related to biomedical research, but this is somewhat different to the area that we are addressing.^19^ In previous work, we have reported that organizations in other countries, which are producing guidance on IP, have systems for consultation, but none appear to have the kind of open system we have described and none have published details about whether consultation results in changes to their intended recommendations.^20^ It is worth emphasizing that NICE's process of public consultation is additional to, and separate from, the involvement of patients and patient organizations in drafting guidance, in ways similar to other health technology assessment organizations.^8^, ^9^, ^10^, ^11^, ^12^, ^13^, ^14^, ^20^ A particular strength of this study is its inclusion of a large number of consultations on very diverse procedures, relevant to a wide range of medical specialties and patient groups. This enhances its capacity to provide insights into the inclination of a wide range of interested parties to respond.

Open consultation on draft recommendations and guidance gives an opportunity for an unlimited range of interested people and organizations to proffer their views and additional information. This supplements input by patients and their representatives during guidance development and provides a valuable check that includes aspects which might previously not have arisen.^21^ The detailed consideration given to each response can be time‐consuming and challenging, but it is feasible and provides an increased level of confidence that published guidance has been open to scrutiny and comment by everyone who might be affected by it. It provides a model which others producing health‐care guidance might wish to consider. When developing systems for public consultation, national guidelines and legal aspects of doing so are important to observe.^3^, ^4^ There are also resource consequences to conducting public consultation robustly, because it requires both technical analyst and administrative support.

Guidance‐producing organizations such as NICE are continually looking at ways of developing and improving the way that they engage with stakeholders and the public. The capacity to garner views and information continues to expand, with the widespread use and evolution of electronic communications and social media. Some detailed reporting by other organizations involved in making health‐care decisions, about their experience with public consultation, would be useful in promoting and developing this agenda. Health services are seeking to become more patient‐centred both at the level of individual care, and also in their health policy decision making. These aims, together with a more general move towards increased responsiveness to stakeholders, make evaluation of these engagements particularly important.

## Ethical approval

Ethical approval was not required as this was a retrospective review of an administrative data set examining numbers of responses and whether changes were made.

## Authorship

BC had the idea for the study. BC and JP designed the study. JTE, HG, VV and LM conducted all the data extraction and analyses. AA reviewed the literature. All authors contributed to the interpretation of findings and the drafting and approval of the final manuscript. BC is guarantor.

## Source of funding

This study was not funded by any external organization. The authors were working for NICE, or on training placement at NICE, at the time of the study. No funder had any role in devising, conducting, analysing or reporting this study. NICE is the sponsor.

## Competing interests

All authors have completed the ICMJE uniform disclosure form at www.icmje.org/coi_disclosure.pdf and declare the following. BC chaired the NICE independent Advisory Committees on IP and on Medical Technologies, and NICE reimbursed his employing hospital Trust for this work. JTE, HG, VV, LM and JP work for the NICE IP Programme as employees of NICE. AA undertook a public health training placement at NICE. All authors declare they have no other competing interests.

## References

[1] Rowe G , Frewer LJ . Public participation methods: a framework for evaluation. Science, Technology, & Human Values, 2000; 25: 3–29.

[2] Abelson J , Forest PG , Eyles J , Smith P , Martin E , Gauvin FP . Deliberations about deliberative methods: issues in the design and evaluation of public participation processes. Social Science and Medicine, 2003; 57: 239–251. 12765705 10.1016/s0277-9536(02)00343-x

[3] Barratt H , Harrison DA , Raine R , Fulop NJ . Factors that influence the way local communities respond to consultation processes about major service change: a qualitative study. Health Policy (Amsterdam, Netherlands), 2015; 119: 1210–1217. 10.1016/j.healthpol.2015.04.015 PMC4561526 25975768

[4] HM Government . Consultation principles: guidance. Cabinet Office. Published 17 July 2012. Updated 14 January 2016. Available at: https://www.gov.uk/government/publications/consultation-principles-guidance, accessed 06 December 2015.

[5] Australian Government . Guidance Note. Best practice consultation. July 2014.

[6] Litva A , Coast J , Donovan J *et al* ‘The public is too subjective’: public involvement at different levels of health‐care decision making. Social Science and Medicine, 2002; 54: 1825–1837. 12113438 10.1016/s0277-9536(01)00151-4

[7] Conklin A , Morris Z , Nolte E . What is the evidence base for public involvement in health‐care policy?: results of a systematic scoping review. Health Expectations, 2015; 18: 153–165. 23252574 10.1111/hex.12038 PMC5060770

[8] van de Bovenkamp HM , Trappenburg MJ . Reconsidering patient participation in guideline development. Health Care Analysis, 2009; 17: 198–216. 19101804 10.1007/s10728-008-0099-3 PMC2725277

[9] Boivin A , Currie K , Fervers B *et al* Patient and public involvement in clinical guidelines: international experiences and future perspectives. Quality and Safety in Health Care, 2010; 19: e22. 10.1136/qshc.2009.034835 20427302

[10] Kelson M , Akl EA , Bastian H *et al* on behalf of the ATS/ERS ad hoc committee on integrating and coordinating efforts in COPD guideline development. Proceedings of the American Thoracic Society, 2012; 9: 262–268. 23256169 10.1513/pats.201208-061ST

[11] Mockford C , Staniszewska S , Griffiths F , Herron‐Marx S . The impact of patient and public involvement on UK NHS health care: a systematic review. International Journal for Quality in Health Care, 2012; 24: 28–38. 22109631 10.1093/intqhc/mzr066

[12] del Campo PD , Gracia J , Blasco JA , Andradas E . A strategy for patient involvement in clinical practice guidelines: methodological approaches. BMJ Quality and Safety, 2011; 20: 779–784. 10.1136/bmjqs.2010.049031 21460393

[13] Eccles M , Grimshaw JM , Skekelle P , Schunemann HJ , Woolf S . Developing clinical practice guidelines: target audiences, identifying topics for guidelines, guideline group composition and functioning and conflicts of interest. Implementation Science, 2012; 7: 60. 22762776 10.1186/1748-5908-7-60 PMC3523009

[14] Abelson J , Giacomini M , Lehoux P , Gauvin F‐P . Bringing, “the public” into health technology assessment and coverage policy decisions: from principle to practice. Health Policy, 2007; 82: 37–50. 16996637 10.1016/j.healthpol.2006.07.009

[15] Siegel JE , Heeringa JW , Carman KL . Public deliberation in decisions about health research. AMA Journal of Ethics, 2013; 15: 56–64. 10.1001/virtualmentor.2013.15.1.pfor2-1301 23356809

[16] Boivin A , Lehoux P , Burgers J , Grol R . What are the key ingredients for effective public involvement in health care policy decisions? A randomised trial process evaluation. The Milbank Quarterly, 2014; 92: 319–350. 24890250 10.1111/1468-0009.12060 PMC4089374

[17] Lowson K , Jenks M , Filby A , Carr L , Campbell B , Powell J . Examining the implementation of NICE guidance: cross‐sectional survey of the use of NICE interventional procedures guidance by NHS Trusts. Implementation Science: IS, 2015; 10: 93. 26122560 10.1186/s13012-015-0283-4 PMC4486420

[18] Campbell B , Morris R , Mandava L *et al* Identifying and selecting new procedures for Health Technology Assessment: a decade of NICE experience in the United Kingdom. International Journal of Technology Assessment in Health Care, 2014; 30: 1–7. 25412656 10.1017/S0266462314000415

[19] Lander J , Hainz T , Hirschberg I , Strech D . Current practice of public involvement activities in biomedical research and innovation: a systematic qualitative review. PLoS ONE, 2014; 9: e113274. doi:10.1371/journal.pone.0113274. 25469705 10.1371/journal.pone.0113274 PMC4254603

[20] Plumb J , Campbell B , Lyratzopoulos G . How guidance on the use of interventional procedures is produced in different countries: an international survey. International Journal of Technology Assessment in Health Care, 2009; 25: 124–133. 19366495 10.1017/S0266462309090175

[21] Campbell B , Chambers E , Kelson M , Bennett E , Lyratzopoulos G . The nature and usefulness of patient experience information in producing guidance about interventional procedures. Quality and Safety in Health Care, 2010; 19: 1–6. 10.1136/qshc.2010.040626 21127090

